# Resting energy expenditure in children and adolescents with cerebral palsy: accuracy of available prediction formulas and development of population-specific methods

**DOI:** 10.3389/fped.2023.1097152

**Published:** 2023-08-23

**Authors:** Barbara Borsani, Giacomo Biganzoli, Francesca Penagini, Alessandra Bosetti, Erica Pendezza, Veronica Perico, Elia Biganzoli, Elvira Verduci, Gian Vincenzo Zuccotti

**Affiliations:** ^1^ Department of Pediatrics, “V. Buzzi” Children’s Hospital, University of Milan, Milan, Italy; ^2^ Medical Statistics Unit, Department of Biomedical and Clinical Sciences L. Sacco, “Luigi Sacco” University Hospital, Università degli Studi di Milano, Milan, Italy; ^3^ Department of Clinical Sciences and Community Health & DSRC, University of Milan, Milan, Italy

**Keywords:** indirect calorimetry, resting energy expenditure, cerebral palsy children, prediction formulae, population-specific methods

## Abstract

**Introduction:**

Energy requirements are difficult to estimate in children with cerebral palsy (CP). Resting energy expenditure (REE), necessary to implement personalized nutritional interventions, is most commonly estimated using prediction formulae since indirect calorimetry, the reference method, is not available in all nutrition units. The aims of the present study were: (1) to evaluate the accuracy of the most commonly used REE prediction formulae developed for healthy children, in children with CP; (2) to assess the accuracy of the REE population-specific formula for CP children proposed in our preliminary report; (3) to develop new population-specific methods.

**Methods:**

REE was measured by indirect calorimetry in 100 children and adolescents with spastic quadriplegic cerebral palsy (SQCP) and estimated on the basis of predictive formulas selected by the clinicians [World Health Organization (WHO), Harris-Benedict, Schofield weight, Schofield weight & height, Oxford, Mifflin formulae and a population-specific formula for CP children developed in our preliminary report].

**Results:**

100 children with SQCP (35 girls, 35%) classified as level V according to gross motor function classification system (GMFCS-V); 64% with oral nutrition, 29% total enteral nutrition (nasogastric tube feeding, percutaneous endoscopic gastrostomy, percutaneous endoscopic transgastric jejunostomy) and 7% mixed nutrition. The median (IQR) REE was 41.96 (17.5) kcal/kg/day.

Statistical analysis highlighted a proportional bias between the indirect calorimetry and all considered predictive formulae for REE determination. By studying the relationship between the bias and the mean values of REE, specific conversion equations were obtained. With a pre-specified model having as predictors the variable weight and the variable Triceps Skinfold (TSF) and, as response the variable REE measured by indirect calorimetry, a predictive nomogram was developed to estimate the REE in this population of children.

**Conclusions:**

We suggest using predictive formulae for healthy children with caution, and where possible carrying out indirect calorimetry to assess REE in children with CP. However, we propose a new tool which could be developed to become an additional help for assessment of REE in the clinical practice.

Future objectives will be to obtain a larger sample size, in a multicenter perspective study, to build a specific predictive model for the REE of the studied population.

## 1. Introduction

The assessment of energy requirements in children with cerebral palsy (CP) is generally difficult since this subgroup of patients has peculiarities such as different growth pattern, body composition, muscle tone, mobility and reduced food intake compared to healthy children. To date, there are no specific recommendations in this field for these children (1–3).

A personalized nutritional intervention is essential to prevent both under and over-nutrition, as both of these conditions have a negative impact on linear growth, peripheral circulation, wound healing, spasticity, irritability, respiratory and gastro-intestinal functions with increased morbidity and reduced quality of life (4). Feeding difficulties encountered in children with oral-motor impairment and CP have been well described (5). However, also overfeeding in children with CP fed via gastrostomy tube (GT) can be possible, with endocrinological and metabolic effects (6).

The cornerstone of a personalized nutritional intervention is the determination of basal metabolic rate (BMR), the amount of energy needed for maintaining vital processes of the body not including activity and food processing. It needs to be assessed in order to evaluate daily energy requirements or total energy expenditure (TEE) (7, 8). Ideally, resting energy expenditure (REE) measured by indirect calorimetry (IC) under strict standard conditions in subjects who have been fasting for at least 8–12 h, can be assumed as BMR.

Nevertheless, the quality of any IC measurement is influenced by a variety of factors in clinical setting, for instance agitation and involuntary muscle movements, conditions that are known to be common among children with CP; therefore, we measured the REE (1, 2).

In common clinical practice REE is estimated using prediction formulae developed in the general population and not specific for children with specific diseases such as those with CP (9).

Indirect calorimetry, which is the reference method to measure REE, is not available in all nutrition units, due to its high costs and the need for specialized personnel. Moreover, the use of IC has been limited since the beginning of the ongoing Covid-19 pandemic (10).

Previous studies have demonstrated that children with CP have a REE generally lower compared to that of healthy children with similar age and weight (6, 11, 12). Energy requirements are related to functional capacity and decrease proportionally with disease severity (12, 13). The degree of functional impairment influences energy requirements; in particular, children dependent on a wheelchair usually require 60%–70% energy, compared to healthy children with the same age and weight (12, 14).

### 1.2. Aims

The aims of the present study were therefore: (1) to evaluate the accuracy of the most commonly used REE prediction formulae developed for healthy children in a sample of children with CP (*n* = 100); (2) to evaluate the accuracy of the REE population-specific formula for CP children developed in our preliminary report; (3) to explore the possibility to develop new population specific method of the assessment of REE.

## 2. Materials and methods

### 2.1. Study participants

We performed a cross sectional study. 100 patients with SQCP classified as level V according to Gross Motor Function Classification System (GMFCS) aged 1–19 years were consecutively evaluated among children referred to the Outpatient Nutrition Clinic of the “Vittore Buzzi” Children's Hospital (Milan, Italy) to perform routine nutritional assessment. Inclusion criteria were diagnosis of SQCP based on a neurological examination by a pediatric neurologist, oral, mixed or tube feeding and spontaneous breathing; while exclusion criteria were the presence of known metabolic disorders, thyroid disease, genetic syndrome, chromosomal abnormalities, medication that altered body composition or energy metabolism and acute or intercurrent diseases.

Oro-motor dysphagia was classified according to the Eating and Drinking Ability Classification System (EDACS levels) (15).

Written informed consent for participation into the study was obtained from the parents or legal guardians of all the patients. The study was approved by the Institutional Review Board (protocol number n. 2021/ST/207. Protocol register n. 0016834, 04/04/2022, CE Area 1 Milan, Italy). All patients underwent full nutritional evaluation including measurement of REE by means of indirect calorimetry.

### 2.2. Nutritional assessment

Weight, length (children <2 years of age) and triceps skinfold (TSF) were measured following international guidelines (16). Weight was measured using a wheelchair scale (Soehnle 7,808 digital multifunction scale). Height (children ≥2 years) was estimated from knee height (KH), measuring the ulnar length with a caliper (GIMA, Italy). Height was calculated using equations [KH: S = (2.69 × KH) + 24.2] (17).

TSF was measured using a skinfold caliper (Holtain T/W skinfold caliper) on the non-dominant or less asymmetrical side of the body. Body mass index (BMI) was calculated as weight (kg)/length or height (m)^2^. Standard deviation scores (SDS) of weight, length, height, weight-for-length, weight-for-height, BMI and TSF were calculated using the WHO reference data (18). WHO standard deviations (SDS) could be calculated for the following intervals of age and anthropometric-parameters: (1) weight-for-age for age group 0–10 years; (2) height-for-age for age group 2–18 years; (3) BMI for-age for age group 2–18 years; (4) arm circumference-for-age for age group 0.25–5 years; (5) triceps skinfold-for-age for age 0.25–5 years.

### 2.3. Predictive equations

In a preliminary report, carried out on 54 subjects, we found that the most commonly employed prediction formulae developed for healthy children give inaccurate estimates of REE in children with CP. In particular, we have shown that an overestimation of REE occurs more frequently with the consequent risk of overfeeding these subjects. In addition, through 4 regression models we developed a population-specific formula (19).

Among the different independent variables (weight, age, sex and height), the formula that allowed the best prediction of REE was obtained using weight alone as a predictive variable (Coefficient of Determination R2 = 0.60).

The population-specific formula developed for the estimation of REE in pediatric patients with PCI was the following: REE (Kcal/day) = 24 × W(kg) + 380 [W = Weight].

In the present study, REE was estimated using the following formulae: WHO (9), Schofield weight (20), Schofield weight and height (20), Harris–Benedict (9), Oxford (21), Mifflin (22) and population-specific formula for CP children developed in our preliminary report (19). Formulas were selected by referring to pediatric guidelines, reviews, and studies on the accuracy of estimation of resting energy expenditure in children and adolescents and are the most commonly used in clinical practice (8, 9, 23). All equations are derived from pediatric populations except for Mifflin. The Mifflin equation was derived using both lean and obese adult subjects but was chosen due to reduced lean mass in children with neurological impairment (24). Table 1 provides the predictive formulas selected by the clinicians to be compared with the measured REE value.

**Table 1:** Formulae used to calculate REE. Rearranged from Carpenter A. et al. (7), Koletzko B. et al. (9) and Rodriguez G. et al. (21).

Source	Age	Gender	Equation
WHO	<3 years	Males	REE = 60.9 wt – 54
		Females	REE = 61.0 wt – 51
	3–10 years	Males	REE = 22.7 wt + 495
		Females	REE = 22.5 wt + 499
	10–18 years	Males	REE = 17.5 wt + 651
		Females	REE = 12.2 wt + 746
	18–30 years	Males	REE = 15.3 wt + 679
		Females	REE = 14.7 wt + 496
Schofield weight	<3 years	Males	REE = 59.5 wt – 30.4
		Females	REE = 58.3 wt – 31.1
	3–10 years	Males	REE = 22.7 wt + 504.3
		Females	REE = 20.3 wt + 485.9
	10–18 years	Males	REE = 17.7 wt + 658.2
		Females	REE = 13.4 wt + 692.6
	18–30 years	Males	REE = 15.0 wt + 692.1
		Females	REE = 14.8 wt + 486.6
Schofield weight and height	<3 years	Males	REE = 1.67 wt + 1,517 ht – 618
		Females	REE = 16.2 wt + 1,023 ht – 413
	3–10 years	Males	REE = 19.6 wt + 130 ht + 415
		Females	REE = 17 wt + 162 ht + 317
	10–18 years	Males	REE = 16.2 wt + 137 ht + 516
		Females	REE = 8.4 wt + 466 ht + 200
	18–30 years	Males	REE = 15.0 wt – 10 ht + 706
		Females	REE = 13.6 wt + 283 ht + 98
Harris-Benedict	All	Males	REE = 66.47 + 13.75 wt + 5.0 ht – 6.76 age
		Females	REE = 655.10 + 9.56 wt + 1.85 ht – 4.68 age
Oxford	<3 years	Males	REE = 61.0 wt – 33.7
		Females	REE = 58.9 wt – 23.1
	3–10 years	Males	REE = 23.3 wt + 514
		Females	REE = 20.1 wt + 507
	10–18 years	Males	REE = 18.4 wt + 581
		Females	REE = 11.1 wt + 761
	18–30 years	Males	REE = 16 wt + 545
		Females	REE = 13.1 wt + 558
Mifflin	All	Males	REE = 9.99 wt + 6.25 ht – 4.92 age + 5
		Females	REE = 9.99 wt + 6.25 ht – 4.92 age – 161

wt, weight; ht, height.

### 2.4. Indirect calorimetry

REE was measured using an open-circuit indirect calorimeter (Vmax 29, Sensor Medics, Yorba Linda, CA). The indirect calorimetry measures mixed expired gas on a breath-by-breath basis. The O2 sensor is an electrochemical fuel cell, and Co2 sensor is an infrared one. It utilizes a mass flow sensor connected to exhaust port of the ventilator (25).

Indirect calorimetry equipment was calibrated daily by the clinician. The calibration was manually performed using the calibration syringe connected to the mass flow sensor using the calibration adapter. Gas analyzers were calibrated prior to each measurement against standard reference gases (O2-CO2) know concentration (Cal1 4% Co2 and 16% O2, Cal2 26% O2). The equipment was located in a silent and thermo-neutral room and the measurement were performed according to the following standard conditions: subjects fasting from at least 12 h (post-absorptive state), mental and physical rest, supine comfortable position with the use of ergonomic pillows, no change in the medication administered in the previous days, not sleeping, with a physiological body temperature and in absence of acute diseases. All subjects were fasting overnight (for 12 h). In order to avoid prolonged and repeated fasting, the indirect calorimetry was scheduled in the same morning of biochemical tests, which had to be performed independently from our study.

A canopy was positioned around the patient's head and the expired air was drawn from the hood at a fixed rate (26). Minimum leakage of the ventilator circuit tube was ensured during measurement, the system provide a display of oxygen, carbon dioxide, and flow to permit direct monitoring of lack detection, FI02 stability and system functioning.

To obtain reliable REE measurement steady state achievement was necessary. Steady state was defined as at least 5 min with <5% variation in respiratory quotient (RQ), <10% variation in oxygen consumption, and <10% variation in minute ventilation (27). After the steady state was reached, the REE measurement was performed for at least 20 min. REE was obtained from oxygen uptake and carbon dioxide output using Weir's equation (28).

The principle that nitrogen is neither utilized nor produced during respiration has enabled the use of formulae minimally deviating from the Weir's equation, in which nitrogen is not considered. Providing that the difference between the results of the complete and abbreviated Weir formulas is less than 2%, we used the abbreviated Weir formula due to the difficulties associated with a 24-hour urine collection which is time-consuming and requires technical and human resources (28).

### 2.5. Nutrient intake

Nutrient intake was estimated using a prospective three-day food dietary record. An expert dietitian/nutritionist instructed the family members or caregivers on how to complete the dietary record for two non-consecutive working days and one weekend day. Every food item or beverage consumed during the day had to be reported, specifying the time and the amount consumed, along with a detailed description of the food item. When was not possible to weight food or beverage, caregivers were trained to quantify them using common kitchen utensils, such as spoons, glasses, or bowls. For children with enteral nutrition, the type of nutritional formula and the amount was recorded. Once returned, every three-day dietary record was checked for completeness by two independent dietitians/nutritionists. Energy, macronutrient intake (protein, carbohydrates, and fats) and micronutrient intake (vitamins and minerals) were estimated using the three-day dietary record and by using the Metadieta software (Me.Te.Da. S.r.l., San Benedetto Del Tronto, Italy).

### 2.6. Statistical analysis

All the continuous variables are reported as median and interquartile range (IQR). According to the interquartile definition, the range is computed considering the 1° and 3° quartile of the distribution (25 percentile-75 percentile). Discrete variables are reported as the number and proportion of subjects with the characteristic of interest. Bland-Altman plots of bias (estimated REE and measured REE) vs. mean [(estimated REE and measured REE)/2] were used to assess the presence of proportional bias. The association between the bias and the mean was assessed using Pearson's product-moment correlation coefficient. Since proportional bias was detected in all cases, the Bland-Altman limits of agreement were computed by considering the procedure proposed by Carstensen (29) when two methods with one measurement on each are to be compared and the presence of proportional bias is assessed. Briefly, if it is observed that the assumption of constant difference between methods is violated, i.e., if there is clear slope in the Bland–Altman plot, the differences (Di) can be regressed on the averages (Ai) [Di = a + bAi + ei, var(ei) = σD2] and the results of the regression (a, the intercept; b, the regression coefficient; t, the residual standard deviation) can be used to derive LoA using this equation: a + bAi ± 2tD or to convert them into prediction intervals of the difference between two future measurements, as follows:

$$\begin{array}{rl} & y_{1|2}=\frac{a}{1-b/2}+\frac{1+b/2}{1-b/2}y_{2}\pm 2\frac{\tau }{1-b/2}\\ & y_{2|1}=\frac{-a}{1+b/2}+\frac{1-b/2}{1+b/2}y_{1}\pm 2\frac{\tau }{1+b/2} \end{array}$$

Then, we modelled the association between the variables clearly associated with the REE starting from a pre-specified model in which REE is the response variables and weight the predictor variable. Through a limited stepwise backward variable selection procedure, starting from all the variable potentially associated to REE in addition to weight (Height, Age, Gender and TSF) we retained in the model the variable triceps skinfold thickness in addition to weight. Non-linear effects were considered by transforming the variable weight, that seemed to be associated non-linearly with the response variable, with a Restricted Cubic Spline with three nodes fixed. For the modelling, only the 54 observations considered in the previous study were used as the training set. The remaining 41 observations were used as the test set.

The adjusted coefficient of determination (R2adj) and the mean squared error of the estimate (MSE) were used as measures of model fit. The 95% confidence intervals of the regression coefficients, R2adj and MSE were calculated using bootstrap on 3,000 random samples of 54 subjects. We then validated the prediction accuracy of the model by considering the Lin's Concordance Correlation Coefficient (CCC) between the predicted values by the new model and the values measured by the indirect calorimetry for the 41 observations of the test set. The concordance correlation coefficient combines measures of both precision and accuracy to determine how far the observed data deviate from the perfect concordance line (i.e., the 45-degree line on a square scatter plot). Lin's coefficient increases in value as a function of the proximity of the reduced major axis of the data to the perfect concordance line (the accuracy of the data). The CCC was compared with the others CCC of the predictive formulas considered to estimate REE.

Finally, to easily describe and visualize the effect size of each predictor retained in the final model on the response variable we produced a nomogram. This could be also useful in clinical practice to easily estimate the REE starting from the variables Weight and TSF.

## 3. Results

### 3.1. Clinical characteristics of the study population

Study population characteristics at baseline are showed in Tables 2, 3.

**Table 2:** Subjects’ characteristics at baseline.

	*n*	Median [IQR] or [*n* %]
Age (years)	100	9 (6;14)
Sex		
Female	100	35 (35%)
Male		65 (65%)
Nutrition		
Oral	100	64 (64%)
Enteral		29 (29%)
Mixed		7 (7%)
Medications		
Baclofen	92	20 (21,7%)
Lioresal		13 (14%)
No treatment		59 (64,1)

**Table 3:** Subjects’ characteristics at baseline.

Overall		Overall		Overall	
(age 0–19 years)		(age 0–5 years)		(age 5–19 years)	
*n*	100	*n*	15	*n*	85
Weight (kg)	19.45 [14.43, 26.05]	Zwei	−0.42 [−2.33, 0.17]	Zwfa	−3.35 [−4.63, −2.05]
[median (IQR)]		[median (IQR)		[median (IQR)]	
Height (cm)	118.00 [107.55, 135.25]	Zlen	−1.28 [−2.09, −0.48]	Zhfa	−2.64 [−3.55, −1.82]
[median (IQR)]		[median (IQR)]		[median (IQR)]	
BMI (kg/m^2^)	13.76 [12.13, 16.24]	Zbmi	−0.78 [−2.48, 1.36]	Zbfa	−2.17 [−4.06, −0.92]
[median (IQR)]		[median (IQR)]		[median (IQR)]	
Weight for length (kg/cm)	–	Zwfl	−1.10 [−2.59, 1.29]	–	–
		[median (IQR)]			
TSF (mm)	7.70 [5.40, 9.20]	Zts	−0.05 [−0.86, 0.53]		
[median (IQR)]		[median (IQR)]			
arm_circumference (cm) [median (IQR)]	18.00 [15.20, 20.00]	Zac	−0.21 [−1.17, 0.62]		
		[median (IQR)]			
REE (kcal/kg/day)	41.96 [32.13,49.6]		46.565 [43.696, 47.6 ]		40.536 [31.45, 49.652]
[median (IQR)]					
REE (kcal/day)	806.50 [648.00, 1,051.50]		610 [529, 638]		878 [719.5, 1,091]
[median (IQR)]					
RQ (respiratory quotient)	0.7144 [0.6639, 0.8025]		0.74 [0.694, 0.83]		0.71 [0.66, 0.79]
[median (IQR)]					

For 0–5 years: BMI-for-age (zbmi); weight-for-length (zwfl); weight-for-age (zwei); height-for-age (zlen); arm circumference (zac); triceps skin fold (zts). For 5–19 years: BMI-for-age (zbfa); weight-for-age (zwfa); height-for-age (zhfa); TSF, triceps skinfold thickness; REE, resting energy expenditure.

According to the GMFCS, based on the assessment of the subject’s motor impairments identifying a five-level scale, our population is classed as level V (GMFCS V) (30, 31).

With regard to the type of movement disorder, 27% of the subjects presented dyskinetic disorder whereas the 43% of the subjects presented spastic disorder.

At the time of the nutritional assessment, the majority of subjects had an oral nutrition (64 subjects, 64% of the total), 29% received a total enteral nutrition by means of a feeding tube (29 subjects, 29% of the total), only 7% received a mixed nutrition (oral + enteral) (7 subjects, 7% of the total).

It is well known that feeding difficulties in these subjects increase as the degree of motor impairment increases.

According to the EDACS level classification (15), the 64 orally fed subjects in the sample were classified as level III, as having eating and drinking restrictions with some safety limitations (the subjects in this group consumed thickened fluids and assumed specific food textures); 7 subjects were classified as level IV, as they had significant limitations in eating and drinking safely (the subjects in this group used a tube for fluid intake and ate orally food with a specific modified consistency), and 29 subjects were classified as level V because they were unable to eat or drink safely and were exclusively enterally fed.

The median (IQR) REE was 806,5 (648; 1,051,5) kcal/day.

With regard to the nutritional status, it can be noted that in our sample undernutrition increases with increasing age (0–5 years SDS weight for length = −1.1; 0–5 years SDS BMI for age = −0.78; 5–19 years SDS BMI for age = −2.17).

One subject with severe chronic malnutrition (SDS BMI for age −4.63) was excluded from the subsequent statistical analysis because the REE value detected by indirect calorimetry (REE 209 kcal/day) would influence the computation of the conversion formulae between the IC and the prediction formulae, and also the model fitting procedure.

**Figure 1:**
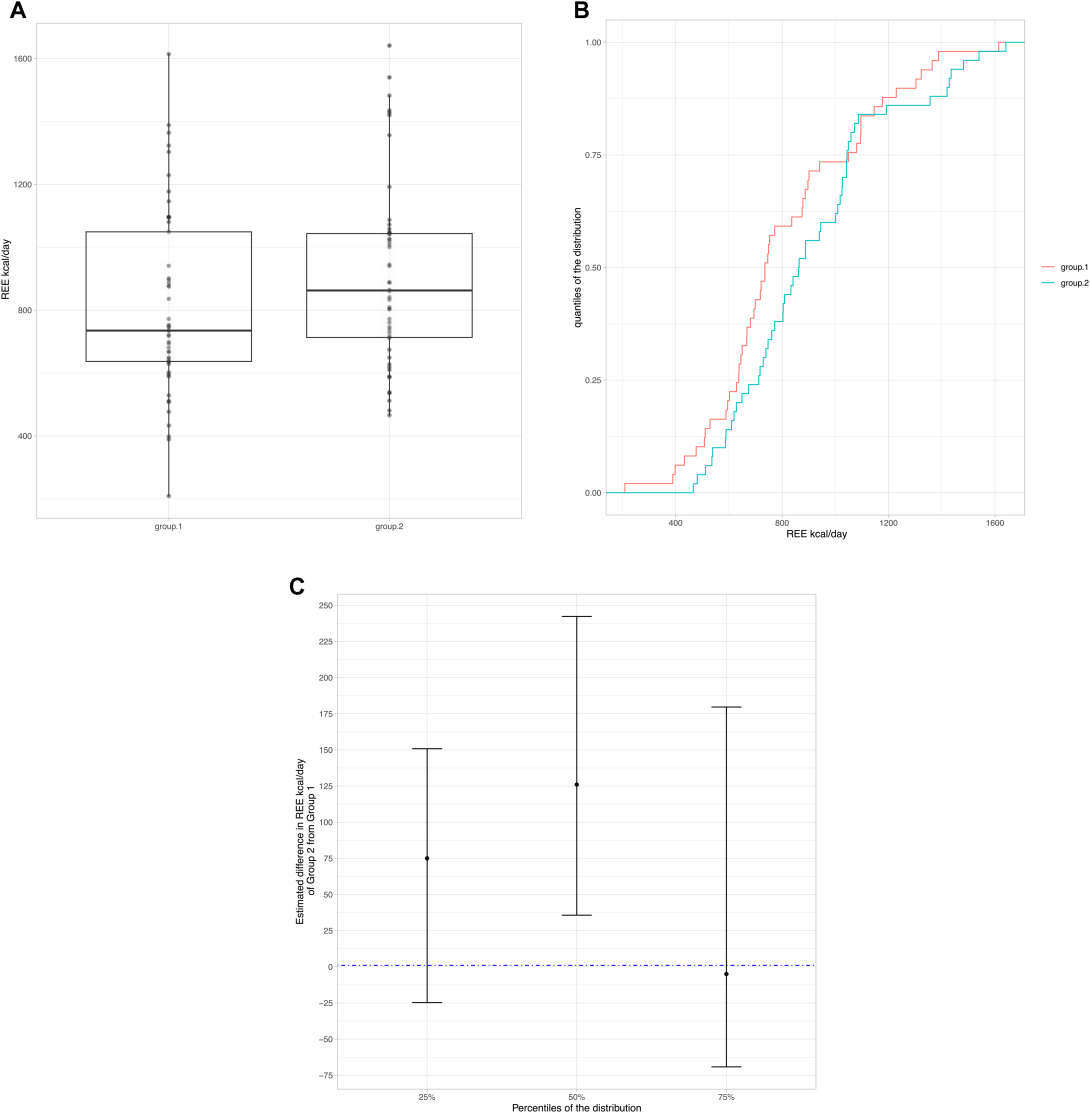
(**A**) Boxplots display the individual REE values represented as points. (**B**) Cumulative distributions of the conditional values to the groups. (**C**) Difference in REE kcal/day values between group 2 and group 1.

To evaluate possible differences in REE we separated the population according to nutritional status by dividing the population into two groups: group 1: moderate + severe malnutrition (*z*-score ≤ −2) and group 2: mild malnutrition + normal nutritional status + overweight/obesity (*z*-score ≥ −1). Boxplots (Figure 1A) display the individual REE values represented as points. Considering the non-normality of the REE kcal/day distributions in the two groups, highlighting differences with box plots alone is difficult, therefore a graph was produced showing the cumulative distributions of the conditional values to the groups (Figure 1B). Group 2, for almost all percentiles of the distribution, tended to have higher REE values than group 1 (curve shifted towards higher REE values). To explore these differences, the 25*, 50* (median) and 75* percentile of the distribution were considered and a regression on the quantiles was applied. The figure (Figure 1C) shows the estimated differences with the respective confidence intervals estimated by the model. There is evidence of a difference in REE kcal/day values between group 2 and group 1 only at 50% (median). At 75% there appears to be no significant difference, however this could simply be determined by a peculiarity of these data.

**Figure 2:**
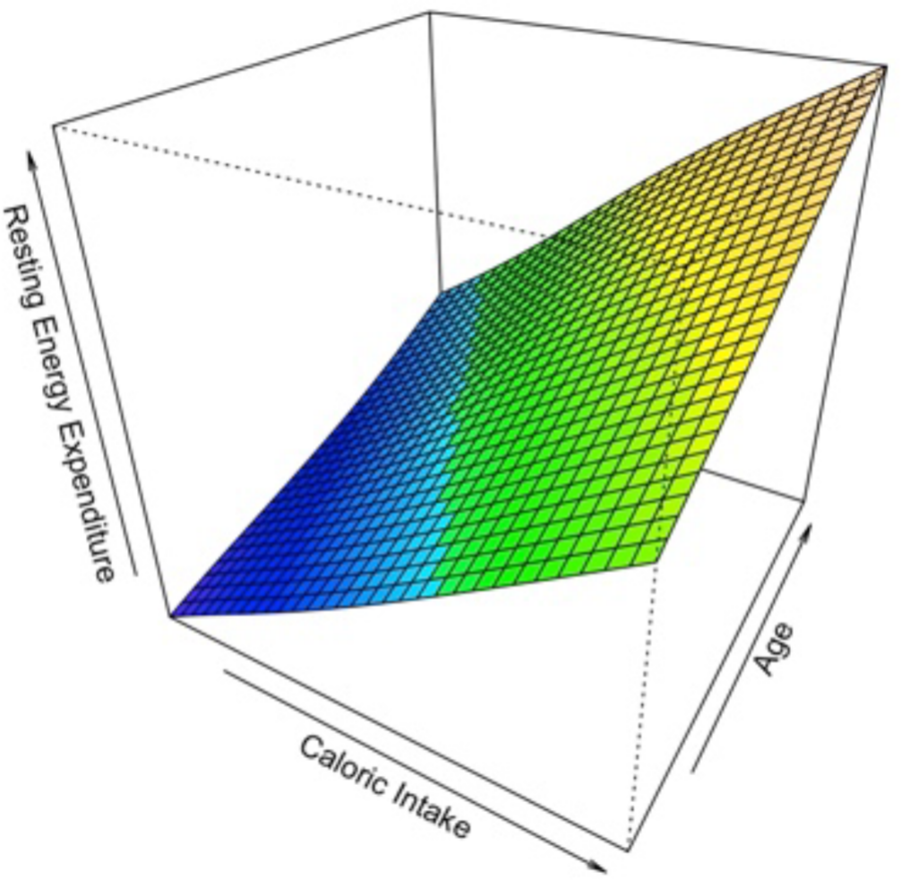
Generalized additive model (GAM).

Among 100 children for which indirect calorimetry REE was measured, only 84 were with complete measurements of Caloric Intake. The median caloric intake was 1,146 Kcal/day (1,000–1,411, 1°–3° quartile respectively). To study the relationship of Caloric Intake vs. the values of Resting Energy Expenditure measured by indirect calorimetry, first a scatterplot has been produced. The relationship between REE and Caloric Intake seemed to be strictly positive monotonic and linear. In a simple linear regression model, the effect of Caloric Intake on the Resting Energy Expenditure was statistically significant. A possible contribution of a non-linear effect for the variable Caloric Intake was tested with a likelihood ratio test, in which the model with the linear term is compared to the model with the additional non-linear term. There wasn't any statistical evidence about the non-linear contribution of Caloric Intake. Then, we adjusted the effect of the variable Caloric Intake in a multivariable linear regression model also considering the variables Age and Gender. The effect of Age showed statistical evidence, while the effect of Gender was not evident. A possible interaction effect for the variable Age and Caloric intake was explored by means of a Generalized Additive Model GAM (Figure 2) in which REE was the response variable and the variables Caloric Intake and Age with a thin plate spline transformation were predictor variables. There wasn't evidence of an interaction effect from the surface plotted. Finally, the multivariable additive model with REE by indirect calorimetry as response variable and Caloric Intake data and Age as predictor variables was considered and showed that with an increase of 388 Kcal/day in the caloric intake, the Resting Energy Expenditure increases by 163 Kcal/Day, at fixed values of Age. With an increase of 4.75 (+1 SD) years of Age, the Resting Energy Expenditure increases by 102 Kcal/Day.

### 3.2. Study of the bias existing between the predictive formulas and the reference method (indirect calorimetry)

**Figure 3:**
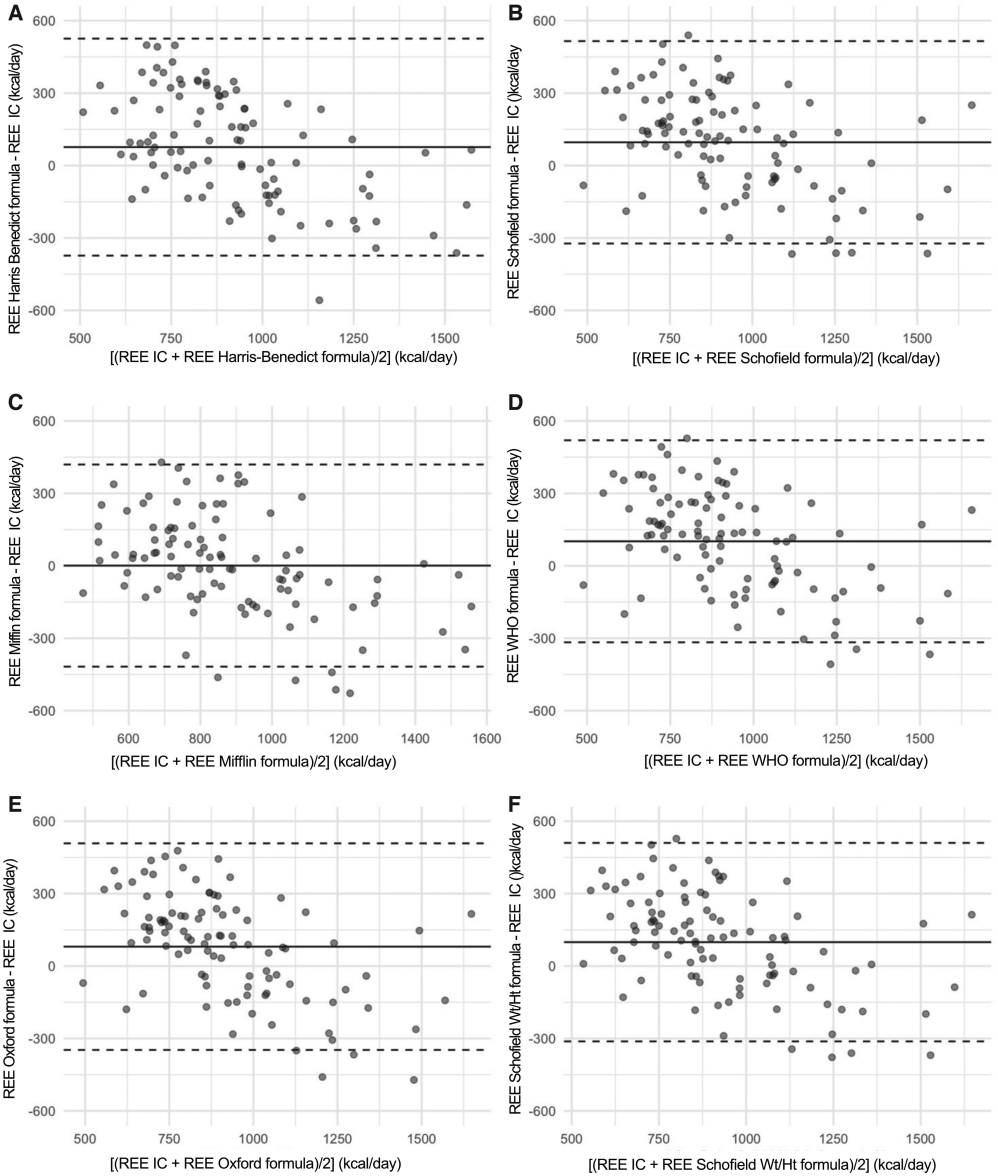
In this figure, the Bland-Altman plot are shown. These are scatterplot that present on the X axis the [(measured REE by Indirect Calorimetry (IC) + estimated REE with formulae)/2] while on the Y axis bias (estimated REE with formulae—measured REE by Indirect Calorimetry (IC). The three horizontal lines represent the mean bias (central line) and its respective Limits of Agreement. It can be noticed that a proportional bias is present, that is the difference changes (with a negative slope) in relation to the increasing the mean values of the REE.

By looking at the Bland-Altman graphs displayed in Figure 3, the bias existing between the reference method (indirect calorimetry) and all the formulas for predicting the REE value is proportional. The difference between the predicted values of the REE by each formula and the actual values measured by indirect calorimetry, is not constant, but changes in relation to the mean values of the REE. In fact, the more the mean value of REE (X axis) increases, the more all the predictive formulas underestimate the actual values of REE, as it is described by the negative slope of the points on the Bland–Altman plots.

**Figure 4:**
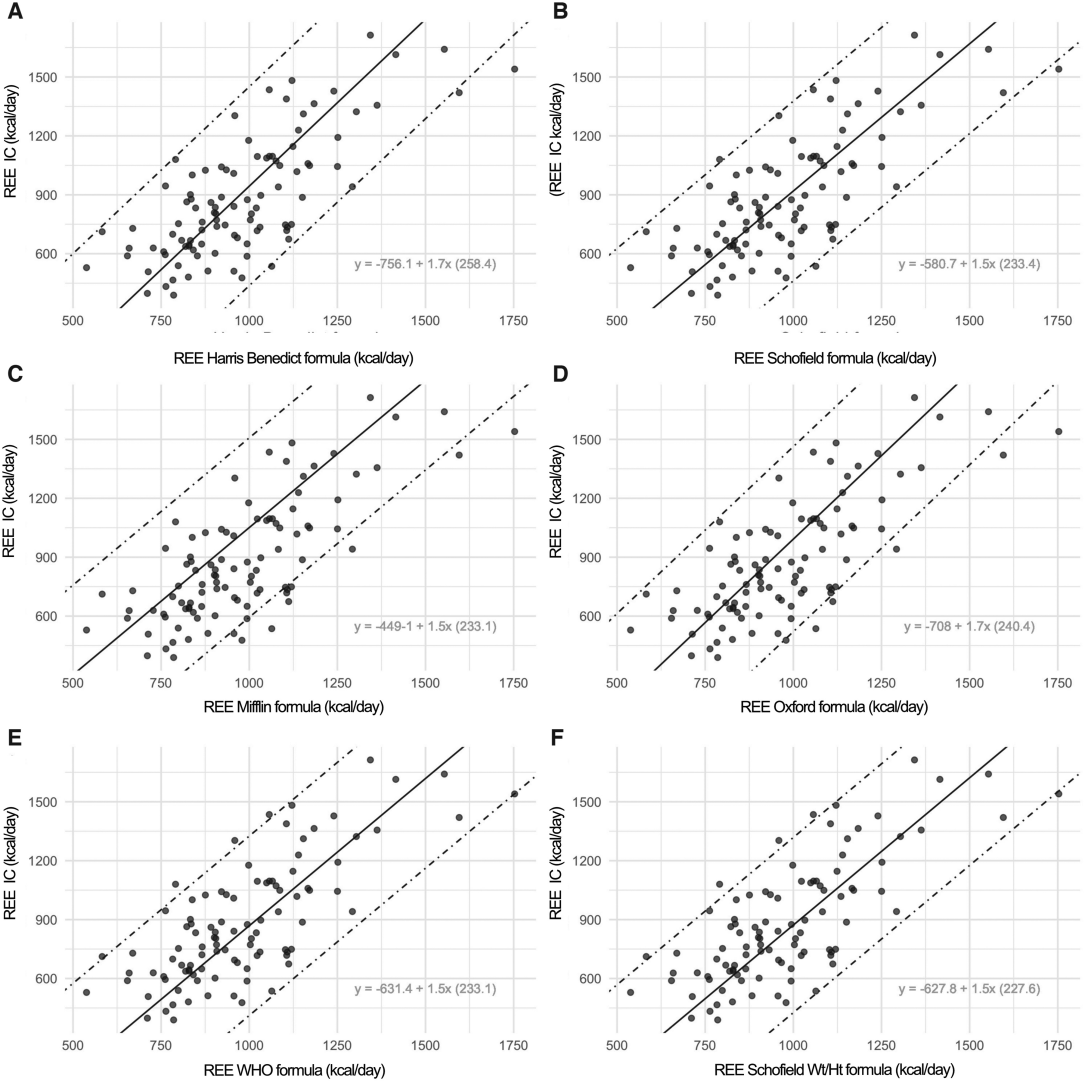
In this figure, the scatterplot displays the prediction limits extracted by rotating the limits of agreement following the negative slope of the bias on the mean values. The prediction intervals defined by the two prediction limits are much more useful because they consistently indicate the range in which a measurement of a specific formula is expected to fall in respect to an Indirect Calorimetry (IC) measurement. Onto the graphs, the specific conversion formulae are displayed. For instance, with an estimated REE value using the Harris-Benedict equation of 956 kcal compared to an REE measured by indirect calorimetry of 873 Kcal, by applying the specific conversion formula: [−756.11 + 1.70(Harris-Benedict)] we obtain a corrected REE value of 870 kcal.

Figure 4 reports the Prediction Limits onto the scatterplot of the indirect calorimetry versus each specific formula.

### 3.3. Development of a population-specific formula

We did not find any statistically evident non-linear effect for the variable weight, as the likelihood ratio test for the non-linear contribution of the variable weight did not show statistical significance. A final multivariable additive linear regression model was considered in which the predictors retained from the limited stepwise backward procedure were TSF, in addition to weight that was a pre-specified variable. Considering the coefficients of the model, the final formula obtained was REE =  + 28.43* Weight −17* TSF + 398.2.

**Figure 5:**
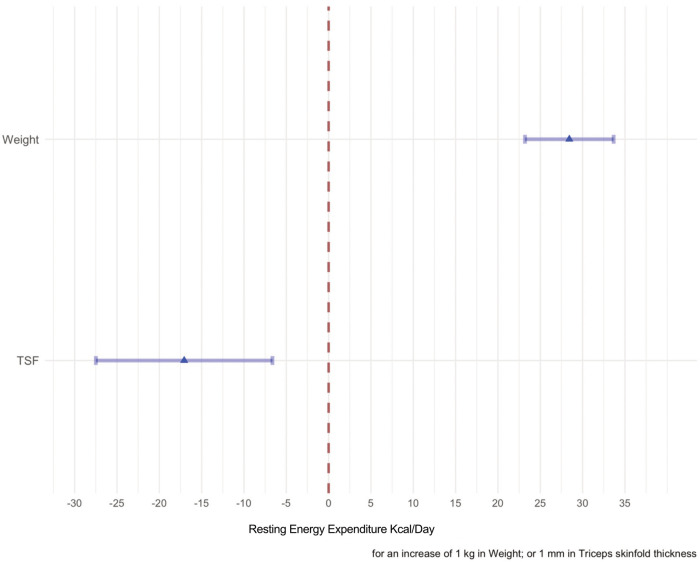
The effects of each predictor on the response variable are shown in this forest plot. The point estimate of the mean change of each predictor when the variable considered in the model varies from the first quartile to the second quartile are represented by the means of a triangle. The shadowed horizontal lines surrounding the triangle are the 95% C.I of the estimate. When weight passes from 14.35–25.5 kg the mean change of the REE is 300 kcal/day, whereas when the TSF passes from 5.6 to 9.2 the mean change is −50 kcal/day.

The effects of the predictors on the REE response variables are displayed in Figure 5.

The R_adj_ and MSE index of a goodness of fit corrected for the optimism are also reported in Table 4.

**Table 4:** Coefficients of determination and mean square error.

	Original index	Training	Test	Optimism correction	Index corrected	*n*
R-square	0.6544	0.661	0.6178	0.0432	0.6112	3,000
MSE	36,310.9501	34,098.920	40,163.5942	−6,064.6744	42,375.6,245	3,000

**Figure 6:**
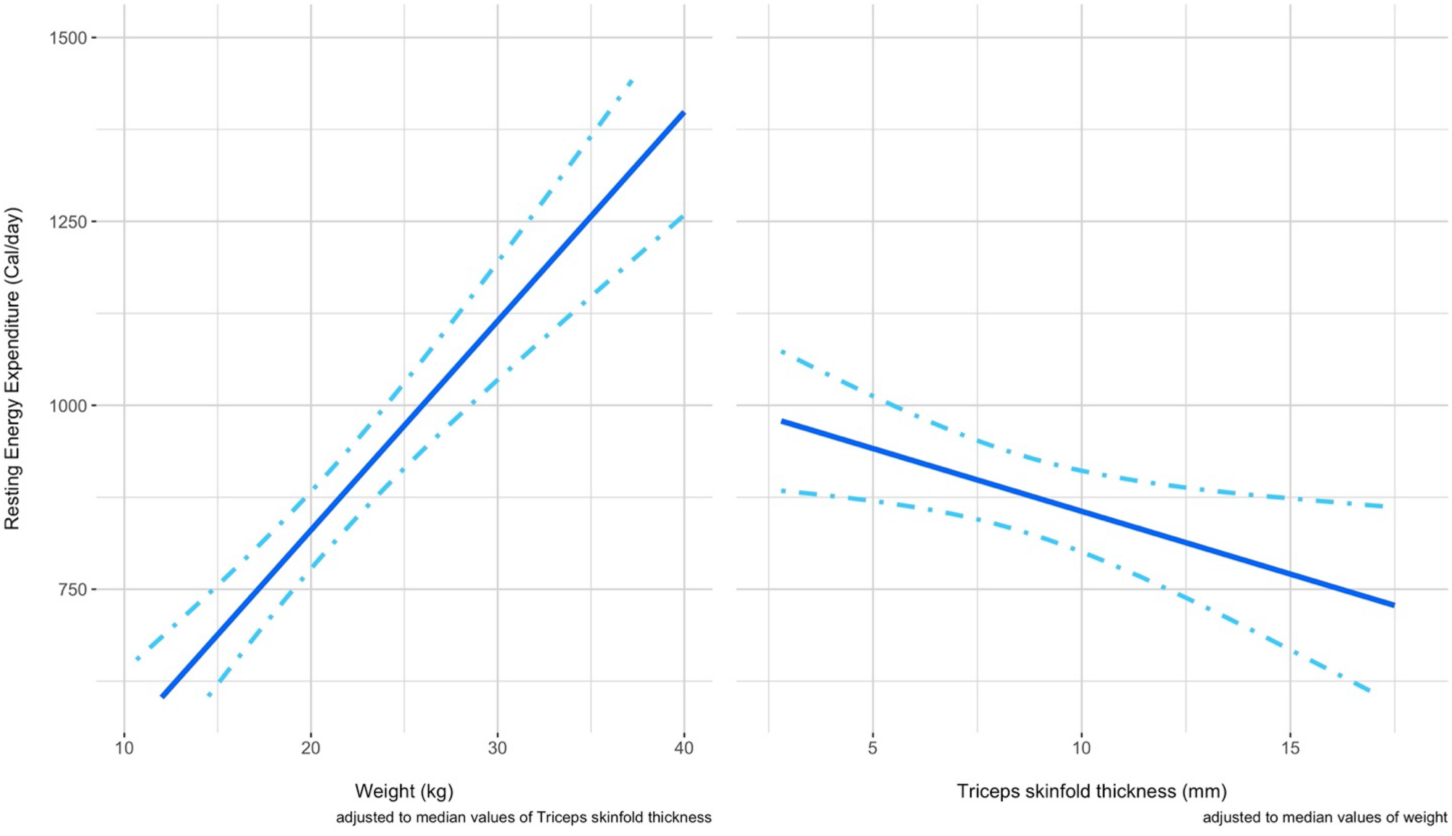
The panel shows in two separated graphs the respective conditional effect of a predictor at fixed values of the other predictor considered. Left panel). The conditional effect of the predictor weight on the REE is shown when a fixed value of TSF is considered (median 7.8 mm) Right panel) The conditional effect of the predictor TSF on the REE is shown when a fixed value of weight (19.7 kg) is considered.

In Figure 6, the conditional effect of each variable at fixed values (the median) of the other predictor can be visualized by looking at the graph reporting on the Y axis the REE and on the X axis the predictor. The variable weight is positively associated to the variable REE measured by IC, while the variable TSF seems to be negatively associated to the variable REE measured by indirect calorimetry. The dot-dashed lines here represent the 95% confidence interval for the effects.

We then assessed the goodness of the prediction of the REE value for the 41 observations for each predictive formula, including our model, by computing the Lin's Concordance Correlation Coefficient with their respective 95% C.I. (reported in Table 5). We could not consider all the observations of the dataset since 54 of them were used to obtain the Population Specific formula showed in the previous article and also used to train our new model for the prediction of the values of REE. Otherwise, the Lin's CCC could have been inflated.

**Table 5:** Lin ’s CCC.

Formula	estimate	95% C.I, lower limit	95% C.I, upper limit
REE_Harris_Benedict	0.271	0.046	0.47
REE_Schofield	0.493	0.284	0.657
REE_Mifflin	0.614	0.396	0.766
REE_Oxford	0.421	0.208	0.596
REE_WHO	0.445	0.235	0.615
REE_Schofield_Wt_Ht	0.478	0.276	0.64
REE_Population Specific equation	0.496	0.25	0.682
New model	0.64	0.446	0.776

It can be noticed that the best performing predictive formulae are Mifflin equation and the new proposed model.

**Figure 7:**
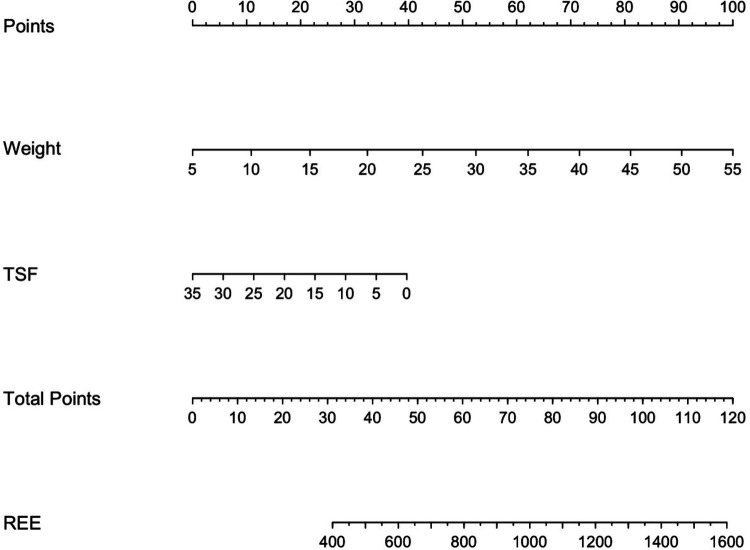
Predictive nomogram for the estimation of the resting energy expenditure in children with PCI when weight and TSF measurements are available. Instructions: locate the measurement of the weight on the respective axis; draw a straight line up to the Points axis to determine how many points are allocated for this variable; repeat this process for the remaining parameter TSF; sum the points and locate this number on the Total points axis; and draw a straight line down to find the REE value predicted.

Finally, a nomogram (Figure 7) was developed. The nomogram can be used to manually obtain predicted values from a regression model and could be also useful in the clinical practice to estimate more accurately the REE when the weight and the TSF values of a child with PCI are available.

## 4. Discussion

This is one of the few studies that assessed the REE in children with CP. The REE of our children is comparable to that of a recently studied population of hospitalized Italian children, consecutively admitted to the Intermediate Care, Nephrology, Intensive Care, Emergency, and Cystic Fibrosis Units (32).

RQ generally ranges between 0.7 and 1, with lower values suggesting an increased oxidation from fats and values tending to 1 indicating prevalently glucidic oxidation. A decreased RQ may be due to several clinical and metabolic conditions including underfeeding, hypoventilation, metabolic alkalosis or hypometabolism, conditions which were common among children in our sample.

With regard to the nutritional status, it can be noted that in our sample undernutrition increases with increasing age (0–5 years SDS weight for length = −1.1; 0–5 years SDS BMI for age = −0.78; 5–19 years SDS BMI for age = −2.17). These results are in agreement with other studies (9, 13).

The proportion of undernourished subjects, according to pediatric malnutrition definition (33), is: moderate + severe malnutrition 52%.

A variation in REE according to nutritional status was found, the group of patients with a more favorable nutritional status had a higher REE value than the malnourished patients (moderate + severe malnutrition).

Moreover, an increase in REE was found on the basis of caloric intake. These data are in agreement with the literature reporting a high prevalence of malnutrition commonly associated with feeding difficulties in children with CP. In general, children with more significant motor impairments have more challenges with oral feeding and have poorer nutritional status (34). This suggest a REE response or adaptation to the poorly nourished condition (12, 35).

In agreement with previous studies (6, 36), our results suggested that the most commonly used predictive formulae, intended for healthy children, often provided inaccurate estimates of REE in children and adolescents with CP. The overestimation of the actual REE may determine an increased risk of over-feeding and metabolic consequences in this population of children.

The formula with the highest accuracy in predicting REE in these subjects was found to be the Mifflin equation. In addition, good performance was also found for the population-specific formula developed in our previous preliminary study (19).

It is interesting to note that the Mifflin equation, the most accurate formula when applied to subjects with severe obesity (BMI > 40 kg/m^2^) (22) in which there is a prevalence of metabolically less active fat mass than lean mass, is derived from a sample of equally distributed (50% and 50%) normal-weight and obese subjects. The improved reliability of this formula could be related to the different body composition, with a lower lean mass, in our GMFCS V population.

Although Mifflin equation was developed for adults, it has been shown to perform well in adolescents with obesity (37). The better performance of Mifflin formulae could be attributed to the presence of proportionately less lean muscle mass in our population as occurs in obese subjects.

Nevertheless, it is relevant to understand whether the performance of the formula is sufficiently accurate to be reliable in clinical practice. Although Mifflin's formula appeared to be the one that agrees most closely with the indirect calorimetry values in addition to the new formula, results displayed a wide range of agreement and consequently it is not sufficiently accurate to recommend its use in clinical practice.

Considering the results of inaccuracy observed on the performance of the predictive formulae, we accordingly suggest an accurate and conscious use of predictive formulae in clinical practice.

Among the new methods explored for estimating the value of REE in these specific population, we can consider the application of conversion formulas reported in each sub-panel of Figure 4, since they can be applied to convert a REE value calculated by a formula into the corresponding value measured by indirect calorimetry, with the corresponding 95% prediction limits.

Furthermore, an association was found between REE, weight and TSF. The new model detected the correlation between REE and lean mass, as for the same body weight a lower TSF value was correlated with a higher REE and vice versa. This result is consistent with data from literature, suggesting a strong correlation between REE and lean mass (38–40).

The TSF is the most commonly measured skinfold, due to its accessibility and prognostic value in malnourished children. A trial assessing malnutrition in children with CP found that a value of TSF <10th centile was able to identify 96% of malnourished children (40).

Arm circumference, in combination with TSF, is a standard component of anthropometric assessment of nutritional status in undernutrition (38) and the assessment of mid-upper arm fat and muscle areas from triceps skinfold and mid-upper arm circumferences measurements can be calculated using several equations (39).

For these reasons, the percentage of fat mass estimated with the equations (i.e., Slaughter et al.) might not be accurate since they are developed for healthy children and they tend to underestimate the value when used in children with CP (1).

Considering that the percentage of body fat mass is an indirectly determined variable, its use in our predictive model could have increased the imprecision of the estimated REE value. Thus, we decided to insert the measured value of TSF, which allowed us to avoid increasing the degree of imprecision of the estimation.

Our new model based on body weight, TSF and REE outperformed the other formulas, showing a highest Lin’s CCC.

Therefore, it was used to develop our nomogram, which may represent a practical tool to be used in clinical routine. It is known that performing an indirect calorimetry is time- and resource consuming, and it requires specific conditions, which make it not performable in all clinical settings.

Clinicians often have to deal with lack of time, equipment (i.e., cot, changing table) or trained staff.

The proposed nomogram is easy and very quick to use, needing only anthropometric assessments which are routinely performed in the clinical practice. Hence, it could represent an useful device whenever performing indirect calorimetry is not possible.

Our study is not without limitations.

Since the exploratory nature of the study, no formal sample size approach was adopted. Thus, a predictive model with reliability could not be built. However, we determined specific conversion equations that are easy to use in clinical practice.

Moreover, reduced peripheral skinfolds may not necessarily correlate to low fat stores, because children with CP tend to store fat more centrally (1). Owing to the difference in fat distribution, the reliability of skinfold measurement is limited (41, 42). This could be one of the possible explanations for the moderate correlation factor for the new model in estimating REE. Another limitation is that, since this study was conducted in a GMFCS V sample, the proposed method should be used only in this population and may not be generalized to all children with cerebral palsy belonging to other GMFCS levels.

Nevertheless, we believe that this tool could offer new help in estimating REE in an easy way, and therefore deserves further investigation. Future objectives will be to obtain a larger sample size, in a multicenter perspective study, to build a specific predictive model for the resting energy expenditure of the studied population, to facilitate the tailoring of energy requirements and optimize the benefits of nutritional intervention by reducing the undernutrition and the risk of over-feeding and metabolic consequences in this population of children.

## 5. Conclusions

There is currently no commonly accepted alternative method other than indirect calorimetry for estimating REE of children and adolescent with CP. We would suggest using predictive formulae for healthy children with caution, and where possible carrying out indirect calorimetry to assess REE in children with CP. However, we propose a new tool which could be developed to become an additional help for assessment of REE in the clinical practice.

## Data Availability

The raw data supporting the conclusions of this article will be made available by the authors, without undue reservation.
